# Spatial epidemiological determinants of severe fever with thrombocytopenia syndrome in Miyazaki, Japan: a GWLR modeling study

**DOI:** 10.1186/s12879-019-4111-3

**Published:** 2019-06-07

**Authors:** Kazuhiro Yasuo, Hiroshi Nishiura

**Affiliations:** 1 Graduate School of Medicine, Hokkaido University, Kita 15 Jo Nishi 7 Chome, Sapporo, Japan; 2 Sapporo Higashi Tokushukai Hospital, 3-1 Kita 33 Jo, Higashi 14 Chome, Sapporo, Japan

**Keywords:** Severe fever with thrombocytopenia syndrome, Ticks, Statistical model, Altitude, Farms, Spatial regression

## Abstract

**Background:**

Cases of severe fever with thrombocytopenia syndrome (SFTS) have increasingly been observed in Miyazaki, southwest Japan. It is critical to identify and elucidate the risk factors of infection at community level. In the present study, we aimed to identify areas with a high risk of SFTS virus infection using a geospatial dataset of SFTS cases in Miyazaki.

**Methods:**

Using 10 × 10-km mesh data and a geographically weighted logistic regression (GWLR) model, we examined the statistical associations between environmental variables and spatial variation in the risk of SFTS. We collected geospatial and population census data as well as forest and agriculture mesh information. Altitude and farmland were selected as two specific variables for predicting the presence of SFTS cases in a given mesh area.

**Results:**

Using GWLR, the area under the receiver operating characteristic curve (AUC) was estimated at 73.9%, outperforming the classical logistic regression model (72.4%). The sensitivity and specificity of the GWLR model were estimated at 90.9 and 58.7%, respectively. We identified altitude (odds ratio (OR) = 0.996, 95% confidence interval (CI): 0.994–0.999 per one-meter elevation) and farmland (OR = 0.999, 95% CI: 0.998–1.000 per % increase) as useful negative predictors of SFTS cases in Miyazaki.

**Conclusions:**

Our study findings revealed that the risk of SFTS is high in geographic areas where farmland area begins to diminish and at mid-level altitudes. Our findings can help to improve the efficiency of ecological and animal surveillance in high-risk areas. Using finer geographic resolution, such surveillance can help raise awareness among local residents in areas with a high risk of SFTS.

**Electronic supplementary material:**

The online version of this article (10.1186/s12879-019-4111-3) contains supplementary material, which is available to authorized users.

## Background

Severe fever with thrombocytopenia syndrome (SFTS) is a fatal tick-borne viral disease that involves thrombocytopenia, caused by the SFTS virus (SFTSV), a phlebovirus in the order Bunyavirales [1, 2]. The disease is characterized by non-specific symptoms, most frequently involving fever, gastrointestinal symptoms, leukopenia, thrombocytopenia, and liver dysfunction, and sometimes involving hemophagocytic syndrome and hemorrhagic tendencies, leading to multiple organ failure [1–4]. The disease was first detected in northeast China in 2007, followed by identification of its common clinical symptoms in 2009 and virus isolation in 2010 [1, 2]. The virus lifecycle and natural mechanisms of sustained transmission (e.g., reservoir animal species) remain to be fully clarified, but transmission via ticks is considered the most plausible route [5–12]. SFTSV has been detected in cats, mice, and wild boars [5, 13–15], and humans are believed to be an accidental host. Although SFTS was initially identified in China, the disease has also been observed in South Korea, Japan, and across all of East Asia [4, 16, 17]. However, the magnitude of cases in Japan has been limited; a total of 391 confirmed cases had been notified in Japan as of 12 December 2018, whereas SFTS cases in China had already numbered in the thousands by the same date.

In recent years, the frequency and geographic areas at risk of tick-borne infectious diseases are known to have expanded in North America, possibly caused by ecological changes of tick abundance that are possibly induced by climate change [18]. Geographic heterogeneity is evident in East Asia as well; for example, the eastern to central mountainous regions of China and Western Japan are known to be focal areas with a higher frequency of SFTS notifications than other regions [4]. Published studies have identified that at altitudes of 80–400 m [15, 19, 20], a number of climatological variables, including average temperature, humidity, and precipitation [10, 15, 19, 21, 22], as well as the density of livestock [7, 8, 10, 13, 17], are associated with an elevated risk of tick-borne infections. Other than published epidemiological studies, the epidemiological data in Japan imply that confirmed cases are frequently seen among farmers who live in rural regions or near forested areas [3, 4, 23]. The case fatality risk (CFR) among confirmed cases was about 30% in 2013 [4]; however, since then, both the CFR and absolute number of deaths have steadily decreased in Japan (e.g., of a total 72 cases in 2018, 3 deaths occurred, with a CFR about 5%) [24].

In Japan, SFTS is designated as a category IV infectious disease according to the Infectious Diseases Law, mandating physicians to notify all laboratory-confirmed cases to the government via the nearest health center [25]. For this reason, all 47 prefectural institutes of public health in Japan are equipped with one-step RT-PCR testing kits, for laboratory confirmation in every prefecture. The collected notification data are sent electronically to the headquarters of the National Institute of Infectious Diseases, and weekly notifications by prefecture are publicly reported. The expected micro-geographic locations of infection are not publicly reported for all of Japan, but suspected locations of infection are reported only among prefectures with high incidence, as described in the Methods below. Moreover, there have been no published epidemiological studies in Japan identifying micro-geographic areas at high risk of SFTSV infection; thus, the underlying mechanisms of infection in geographically high-risk areas have yet to be elucidated. The generally acknowledged method for preventing SFTS is to avoid tick bites by (i) ensuring that there is no exposure, such as by avoiding high-risk areas; (ii) keeping ticks away from exposed skin by wearing long-sleeved shirts, long pants, and high boots; (iii) using insect repellent, such as products containing DEET, on the body; and (iv) checking the entire body for ticks after activities associated with potential exposure. Despite this knowledge, many individuals have considerable difficulty in completely avoiding tick bites owing to their occupation or living circumstances [26, 27].

To develop effective future countermeasures, it is critical to identify and communicate the risk factors of infection, with high geospatial resolution at the community level. As a first step, in the present study we aimed to identify areas with a high risk of SFTSV infection and their characteristics using a geospatial dataset of SFTS cases in Miyazaki, Japan.

## Methods

### Collection of epidemiological data

The present study focused on Miyazaki Prefecture, located on the southeast coast of Kyushu Island, which is in the westernmost part of Japan. Of a total 392 confirmed cases of SFTS, 61 were notified in Miyazaki. From 2013 to 2018, the annual number of confirmed cases in this prefecture was 7, 11, 9, 9, 13, and 12, respectively. Only Miyazaki Prefecture has publicly reported the geographic coordinates of suspected locations of infection, i.e., areas with a high likelihood of receiving a tick bite. This information did not involve history taking of travel or exposure behaviors but instead rested purely on known history of a tick bite. Notified cases are initially suspected of SFTSV infection if patients present atypical clinical symptoms and the following clinical diagnostic conditions: (i) fever greater than 38.0 °C; (ii) gastrointestinal symptoms; (iii) thrombocytopenia (less than 100,000/mm^3^); (iv) leukopenia (less than 4000/mm^3^); (v) elevated alanine aminotransferase, alanine transaminase, and lactate dehydrogenase; (vi) absence of identifiable causes other than tick bites; and (vii) the need for intensive care or fatal outcome [23]. All patients with suspected SFTS undergo one-step RT-PCR testing for confirmatory diagnosis, and only confirmed cases are notified to the government. Essential conditions for notification include laboratory confirmation; even clinically mild cases that do not meet the abovementioned conditions must also be reported [23, 28].

In the present study, we stratified cases according to the year of notification, allowing us to separate patients diagnosed in 2017 from those diagnosed in 2016 and earlier. The earliest year of observation was 2013. Of a cumulative 61 cases in Miyazaki as of December 2018, 34 exact geographic locations were available at the end of 2016, and an additional 8 locations were identified in 2017. Of the total 61 cases, the location of receiving a tick bite remained unknown in 19 cases. Among the 42 cases with a known location where a tick bite occurred, no cases had identical geographic coordinates. We collected datasets of latitude and longitude of the 42 known locations from 2013 to 2017. Patients’ times of illness onset were recorded separately from the location data; therefore, we were unable to match the geographic location of tick bites with the time of illness onset or diagnosis. Of the 34 locations that were notified by the end of 2016, 12 patients were bitten on farms, 6 in the woods, 5 in nearby gardens, 4 while walking or trekking, 2 while landscape gardening, and 1 patient was bitten while hunting; the land-use information of the exposure locations for the remaining 6 patients was not specified.

### Collection of geographic data

We used a 10 *×* 10-km mesh to divide the land area of Miyazaki into 97 square area units of 100 km^2^ each; this is also referred to as second-order mesh statistics. According to mesh areas, we first collected forest and agriculture mesh data from the National Land and Information Division of Japan [29], to identify whether a particular geographic location in Miyazaki is predominantly used for forest or agricultural purposes. The dataset rests on a survey, referred to as the Fundamental Land Classification Survey, which classifies the national land use using standard scientific criteria. The survey data included up to the end of 2015, and the mesh proportion of land use for agriculture and forest only varied within 5% of the value from 2010 to 2015. Second, the altitude of each mesh area was manually obtained from a map of the Geospatial Information Authority of Japan in 2018 [30]. Using altitude data for each center of a 1 *×* 1-km mesh, we collected 100 datasets of altitude for each 10 *×* 10-km mesh, and we took the median value as the altitude representing the 10 *×* 10-km mesh. Third, additional census data of cultivated land in 2015 were collected from the Ministry of Agriculture, Forestry and Fisheries [31]. Using the dataset of cultivated land, we calculated the proportion of farmland in the total mesh area (hereafter referred to as “farmland”). Fourth, regional mesh statistics of population census, including the age distribution, were collected from the census data of Miyazaki Prefecture. Fifth, climatological variables including regional mesh statistics of annual average temperature and average precipitation in 2014 were collected from the Japan Meteorological Agency.

### Statistical analysis

We first plotted the suspected geographic locations of SFTSV infections on a map of Miyazaki, determining the regional mesh to which each case belonged. The original precision of suspected location of tick bite exposure was given using a circle with a diameter of 3 km, with the center of the circle indicating the exact suspected location based on interviews regarding the tick bite history. The diameter of 3 km was due to scaling of the original map, and thus, we used only the geographic information of the center of the circle in the following analyses. Then, mesh areas were classified according to two binary categories, i.e., mesh units with and without one or more cases, which we aimed to predict using environmental and geospatial explanatory variables. Second, we examined univariate associations of the presence of an SFTS case with the following variables: (i) the proportion of elderly residents (aged 65 years or older), (ii) percentage of farmland area, (iii) proportion of abandoned farmland (in which the land was notified as abandoned by farmers to the Ministry of Agriculture, Forestry and Fisheries), (iv) the proportion of forest, and (v) altitude (measured at the center of each mesh area). In addition, to avoid statistical interactions between two or more explanatory variables, we examined the linear correlation between each pair of explanatory variables. Third, a multivariate model was used to address confounding variables with a logistic regression (LR) model. Moreover, to account for the geographically dependent structure, geographically weighted logistic regression (GWLR) was used. GWLR is known to be an effective tool for analyzing geospatially dependent data (or spatial autocorrelation). Neglecting spatial dependence can frequently underestimate the *p*-value of the regression coefficient, resulting in erroneous conclusions about the significance of results [32–39]. Further, GWLR allows for variation of the regression coefficient by mesh area. We let *y*_i_ be the linear predictor of mesh *i*, which is modeled as

1 $$ {y}_i={\beta}_0+\sum \limits_j{\beta}_j{x}_{j,i}, $$

in the LR model, where *β*_0_ is the intercept and *β*_j_ is the coefficient of input *x*_j, i_ of the *j*-th explanatory variable. The logistic function uses *p*_i_ = 1/(1 + exp(−*y*_i_)) as the probability of observing the case in mesh *i*. The GWLR model treats the coefficient *β* as the local (varying) function by location, i.e.,

2 $$ {y}_i={\beta}_0\left({u}_i,{v}_i\right)+\sum \limits_j{\beta}_j\left({u}_i,{v}_i\right){x}_{j,i}, $$

where *u*_i_ and *v*_i_ represent the geographic coordinates of mesh *i*. The function *β* (*u*_i_,*v*_i_) allows us to account for dependence of the parameters in nearby geographic areas; in addition, non-uniformity of parameters across space is permitted. We calculated the Euclidean distance, *d*_ab_, between all pairs of geographic mesh points, *a* and *b*, and modeled the dependence function using the adaptive bi-square model,

3 $$ w\left({d}_{ab}\right)={\left(1-\frac{{d_{ab}}^2}{{\theta_{a(k)}}^2}\right)}^2, $$

where *θ* is the bandwidth of geographic dependence. The adaptive bi-square method was used, because the bi-square model has been described as the standard method for exploration; in addition, the alternative method, i.e., adaptive Gaussian method, exhibited smaller predictive performance during our preparatory calculations.

We optimized both the multivariate LR and GWLR models using JMP version 14 (SAS Institute Inc., Cary, NC, USA) and GWR4 (GWR4 Development Team, Kyoto, Japan), respectively, using selected variables that appeared to consistently describe the observation process of SFTS cases in mesh areas. We performed two different predictions using the LR and GWLR models, to assess their predictive performance. In one procedure, we aimed to predict all observed datasets, using all previously observed datasets from 2013 to 2017 as the learning data. However, it is known that the predictive performance using all observed datasets can sometimes be overly optimistic because that process is not strictly a prediction of the future [40, 41]. To avoid any confusion regarding this matter, we also predicted the incidence in 2017 using data from 2013 to 2016 [41]. In this instance, the future data (2017) were predicted using the past data (2013–2016). As part of sensitivity analyses, we also examined ecological associations not only using 10 *×* 10-km mesh data but also 1 *×* 1-km mesh data with an LR model.

For model comparisons, we used the likelihood ratio test. To assess the predictive performance, we computed the exact 95% confidence interval (CI) of sensitivity and specificity, using the quantile function of the binomial distribution. We also examined the area under the receiver operating characteristic (ROC) curve (AUC), along with the positive predictive value (PPV) and negative predictive value (NPV). We used the Youden index (i.e., sensitivity + specificity − 1) to identify the sensitivity and specificity of LR at an optimal threshold. Calculations of 95% CIs of the PPV and NPV were based on the Wald method, with the PPV and NPV variances determined using the delta method [42]. For calculation of the 95% CI of the AUC, we used the Wald method with logit transformation of the AUC [43, 44]. The R statistical software (R Development Core Team) was used for these analyses.

### Ethical considerations

In the present study, we analyzed data that are publicly available. As such, the datasets used in this study were de-identified and fully anonymized in advance. The analysis of publicly available data with no identifiable information does not require ethical approval.

### Data sources

The occurrence of SFTS cases, altitude, and the proportion of farmland are available as Additional file 1.

## Results

**Fig. 1:**
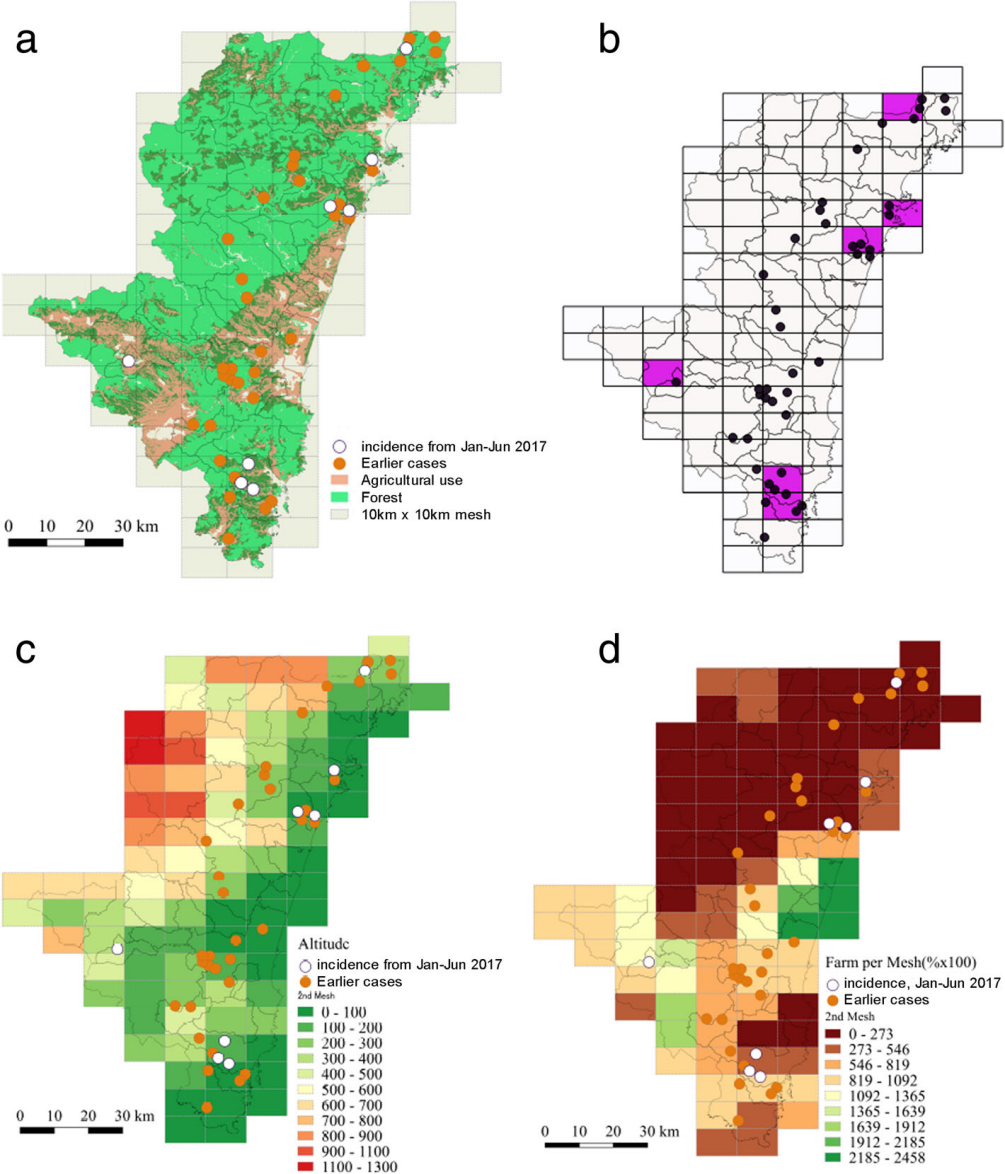
Geographic distribution of severe fever with thrombocytopenia syndrome cases and environmental variables. **a** Geographic areas classified as forest (green) and agricultural land (light brown) overlaid with 42 confirmed cases. **b** Map of Miyazaki Prefecture divided into a total of 97 mesh units consisting of 10 *×* 10-km (second-order mesh) squares. Purple-colored mesh areas represent geographic units with recent cases in 2017. The remaining points represent cases observed during 2013–2016. **c** Altitude and **d** the proportion of farmland in Miyazaki. Altitude was measured at the center of each mesh area. Note that the unit of panel D is percentage times 100. In panels (**a**, **c**, and **d**), cases in 2017 are shown as empty circles; the remaining orange circles represent cases diagnosed during 2013–2016

Figure 1a shows a map of all suspected geographic locations of SFTSV infection (*n* = 42, including 8 locations in 2017). Miyazaki Prefecture faces the Pacific Ocean on its eastern margin, and it borders Oita and Kagoshima prefectures on its northern and southern margins, respectively. The western margin of Miyazaki is a mountainous area bordering Kumamoto Prefecture. The eastern area aligned with the coast is referred to as Miyazaki Plain, where the population density tends to be higher than other areas. Although arbitrary, Fig. 1a indicates that cases of SFTS occurred near rivers, in transitional zones between farms and forest, and at altitudes that were not very elevated but also not close to sea level. Figure 1b shows a comparison of 34 locations at the end of 2016 and an additional 8 locations in 2017. An SFTS case was observed for the first time in 2017 only in mesh units overlapping with Takaharu town, which had no previously reported cases; otherwise, all mesh areas had at least one reported case prior to 2017.

Figure 1c and d shows the altitude and proportion of farmland at the second-order mesh level. Using univariate LR analysis, altitude (*p* < 0.01) was identified as a significant negative predictor of SFTS cases whereas farmland alone (*p* = 0.70), the proportion of elderly residents (*p* = 0.81), proportion of abandoned farmland (*p* = 0.46), and forest ratio (*p* = 0.38) did not have any significant associations with the presence of SFTS. Annual average precipitation (*p* = 0.94) was not significantly associated with the presence of SFTS whereas annual average temperature showed a positive association with SFTS (*p* = 0.05). Nevertheless, the significant association between SFTS and average temperature disappeared when altitude was modeled together with the average temperature and an interaction term of these variables was taken into account (*p* = 0.21 for temperature and *p* = 0.03 for altitude). Among non-significant variables, farmland showed significant correlations with the proportion of abandoned farmland (correlation coefficient, r = 0.75), forest ratio (r = 0.82), and proportion of elderly residents (r = 0.38). Of these, farmland was the variable that excellently reflects the changing land use from farmland to forest, and moreover, the observations depicted in Fig. 1a indicate frequent infections at mid-level altitudes and also in areas between farms and mountains; therefore, owing to the univariate results as well as the correlations among explanatory variables, we used only altitude and farmland as explanatory variables in the subsequent multivariate analyses.

Using altitude and farmland as input, we optimized the multivariate GWLR model. Table 1 shows the global terms of GWLR, revealing that altitude was a significant negative factor of infection. When the data at the end of 2016 were used to predict 2017, both altitude and farmland appeared to act as significant negative predictors. When all existing cases were predicted, GWLR outperformed the LR by means of the likelihood ratio test (*p* = 0.04). Similarly, when 2017 was predicted, the GWLR model significantly reduced deviations from the LR with a *p*-value < 0.001 (likelihood ratio test).

**Table 1:** Estimated odds ratios for cases of severe fever with thrombocytopenia syndrome using geographically-weighted logistic regression

Subject	Variable	Odds ratio (95% Confidence interval)	*p*-value
All cases	Intercept	NA	0.57
	Altitude (m)	0.996 (0.994, 0.999)	*0.004
	Farmland (%)	0.999 (0.998, 1.000)	0.10
Cases by 2016	Intercept	NA	0.40
	Altitude (m)	0.996 (0.993, 0.999)	*0.002
	Farmland (%)	0.999 (0.998, 1.000)	*0.049

All cases represent prediction using all available datasets by the end of 2017. To avoid overly optimistic results, we also predicted 2017 cases using data to the end of 2016 (Cases by 2016). The coefficient of determination, R^2^ using all cases was 0.12. Similarly, the R^2^ with cases at the end of 2016 was 0.14.

Asterisks * before *p*-value indicate significant results

**Fig. 2:**
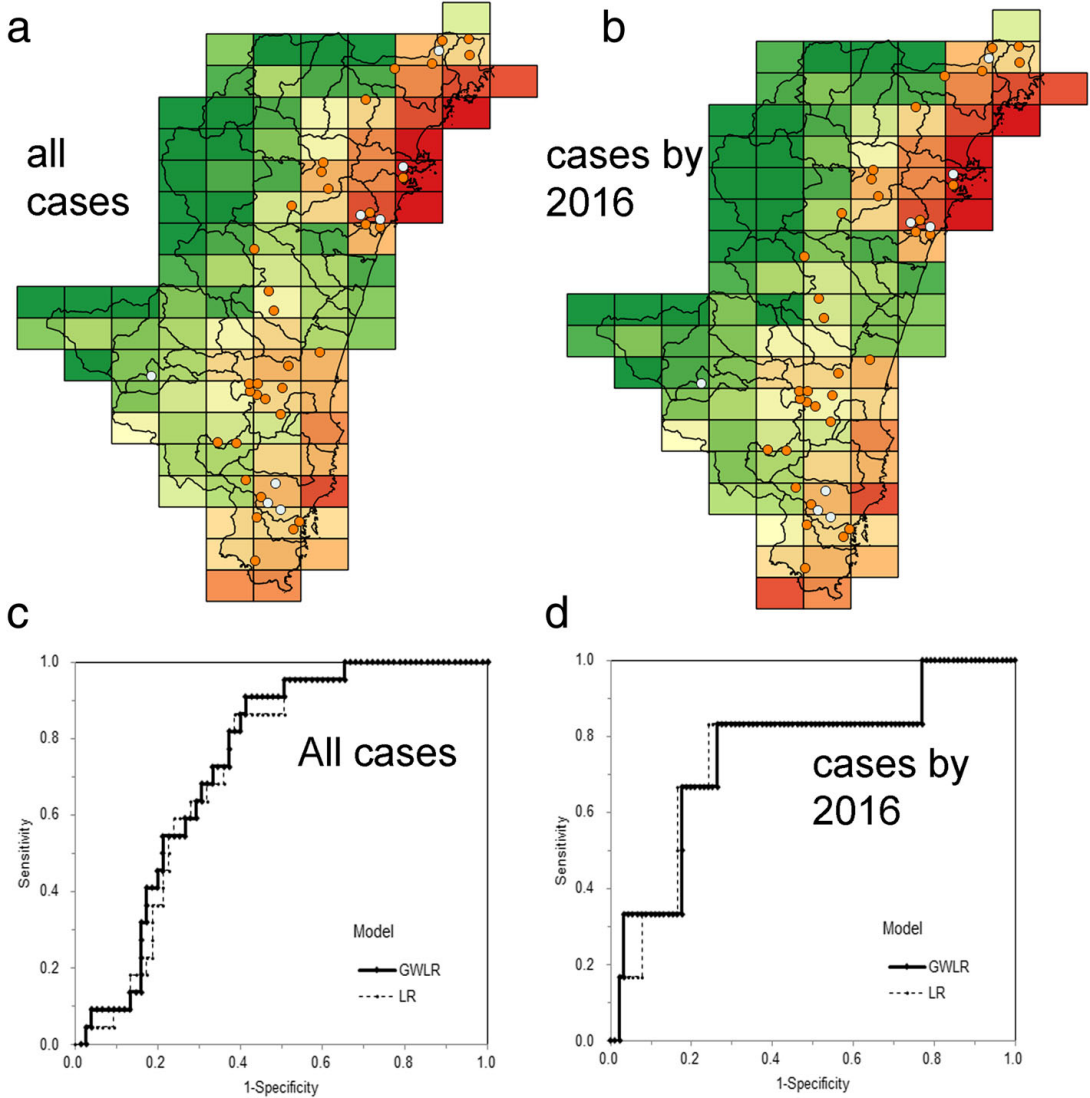
Predicted risk map of severe fever with thrombocytopenia syndrome cases using geographically weighted logistic regression. **a** Geographically weighted logistic regression (GWLR) model to predict all cases and (**b**) cases in 2017. All cases during 2013–2017 were used as the learning data of A; data from 2013 to 2016 were used for B. The color changes from green to red with elevated risk. Low-risk areas below the maximum Youden index value are colored green, and otherwise yellow to red. Observed cases in 2017 are empty circles; the remaining orange circles represent cases diagnosed during 2013–2016. Receiver operating characteristic curves for correctly predicting SFTS cases in Miyazaki using (**c**) the entire dataset and (**d**) data from 2013 to 2016. In addition to GWLR, the results from the logistic regression model are also overlaid

Figure 2a and c shows the predicted risk maps along with the evaluation of predictive performance using the ROC curve. The AUC of GWLR (73.9%) was greater than that of LR (72.4%) (Table 2). Figure 2b and d shows the predicted risk maps with the ROC curve of prediction in 2017. Again, the AUC of GWLR (76.6%) outperformed that of LR (75.6%). Except for the one case in 2017 in Takaharu town, the remaining cases (5 of 6 meshes containing a total of 7 cases) occurred in mesh areas where the risk of SFTS cases was above the median, and the concordance of the estimates with the actual observed locations (empty points) was verified.

**Table 2:** Estimated predictive performance of logistic regression models in severe fever with thrombocytopenia syndrome, Miyazaki Prefecture

All subjects (*n* = 97 mesh)	LR (all cases)	GWLR (all cases)	LR (cases by 2016)	GWLR (cases by 2016)
Proportion (%)	22.7 (14.3, 31.0)	22.7 (14.3, 31.0)	6.2 (1.4, 11.0)	6.2 (1.4, 11.0)
Sensitivity (%)	86.4 (72.0, 100)	90.9 (78.9100)	83.3 (53.5100)	83.3 (53.5, 100)
Specificity (%)	61.3 (50.3, 72.4)	58.7 (47.5,69.8)	75.8 (67.0,84.6)	73.6 (64.6, 82.7)
PPV (%)	39.6 (31.7, 47.5)	39.2 (32.1,46.4)	18.5 (10.8,26.3)	17.2 (10.2, 24.3)
NPV (%)	93.9 (87.7, 100)	95.7 (90.1100)	98.6 (96.0,100)	98.5 (95.9100)
Precision (%)	39.6 (25.7, 53.4)	37.7 (24.7, 50.8)	19.2 (4.1, 34.4)	19.2 (4.1, 34.4)
F1-score	0.54	0.53	0.31	0.31
AUC (%)	72.4 (62.7, 80.3)	73.9 (63.5, 80.9)	75.6 (66.2, 83.1)	76.6 (67.2,83.9)

*Abbreviations*: *LR* Logistic regression, *GWLR* Geographically weighted logistic regression, *PPV* Positive predictive value, *NPV* Negative predictive value, *F1-score* Harmonic average of the precision and recall, *AUC* Area under the receiver operating characteristic curve

Values in parentheses are 95% confidence intervals

All cases represent prediction using all available datasets at the end of 2017. To avoid overly optimistic results, we also predicted 2017 cases using data at the end of 2016 (Cases by 2016)

Using the Youden index, the sensitivity and specificity of LR were estimated at 86.4 and 61.3%, and those of GWLR were 90.9 and 58.7%, respectively (Table 2). The PPV and NPV of LR were 39.6 and 93.9%, and comparable with those of GWLR, estimated at 39.2 and 95.7%, respectively. The actual observed mesh areas in Miyazaki, Nobeoka, and Nichinan cities were in line with high-risk areas, as indicated by the risk maps. In the prediction using only the 2017 data, the sensitivity and specificity of LR were estimated at 83.3 and 75.8%, and those of GWLR were 83.3 and 73.6%, respectively (Table 2). The PPV and NPV of LR were 18.5 and 98.6%, and those of GWLR were comparable, estimated at 17.2 and 98.5%, respectively. Precision and F1-score, given as the harmonic average of precision and sensitivity (=recall), were also comparable between the LR and GWLR models (Table 2).

Table 3 shows the summary statistics of local coefficients for altitude and farmland using the GWLR model. Both altitude and farmland yielded negative coefficient values, indicating that the local coefficients of these two variables always highlighted them as significant negative predictors of SFTS cases. Whereas the coefficients of altitude varied widely, those of farmland only varied slightly among the included mesh areas in Miyazaki. Similar patterns were seen when predicting 2017 data only, but variations in the estimated coefficients for farmland were greater than those predicted for the entire dataset.

**Table 3:** Summary statistics of varying (local) coefficients in geographically weighted logistic regression for SFTS cases, Miyazaki

Parameter	Minimum	25 percentile	Median	75 percentile	Maximum
All cases					
Intercept	0.0839	0.4698	0.6430	0.8163	1.3150
Altitude (m)	−0.0054	−0.0046	−0.0041	−0.0035	− 0.0024
Farmland (%)	− 0.0013	− 0.0010	− 0.0010	− 0.0009	−0.0007
Cases by 2016					
Intercept	0.2790	0.5964	0.7880	1.0626	1.7268
Altitude (m)	−0.0063	−0.0051	−0.0044	− 0.0040	−0.0030
Farmland (%)	−0.0018	−0.0014	− 0.0012	−0.0011	− 0.0009

*Abbreviations*: *LR* Logistic regression, *GWLR* Geographically weighted logistic regression, *SFTS* Severe fever with thrombocytopenia syndrome

All cases represent prediction using all available datasets at the end of 2017

To avoid overly optimistic results, we also predicted 2017 cases using the data at the end of 2016 (Cases by 2016)

As a sensitivity analysis, we examined possible explanatory factors, using 1 × 1-km mesh data and a multivariable LR model. Altitude (*p* < 0.001) and farmland (*p* < 0.02) consistently appeared to be significant explanatory variables, explaining the observed pattern well (AUC = 72.4%).

## Discussion

In the present study, we analyzed the spatial epidemiological risk of SFTS in Miyazaki, Japan, using 10 *×* 10-km mesh data and LR and GWLR models. By exploiting the spatial variation in the risk of SFTS, we attempted to identify environmental risk factors, exploring a number of variables including the proportion of abandoned farmland and the forest ratio, which are possibly associated with the vector ecology and risk of tick bites. From the results depicted in Fig. 1b, we hypothesized that most cases of SFTS in Miyazaki, Nobeoka, and Nichinan cities occur along the lower reaches of rivers at mid-level altitudes in locations between farmland and forest. Accordingly, we restricted the explanatory variables to altitude and farmland and identified these two variables as useful negative predictors of SFTS cases in Miyazaki.

Altitude has been identified as an important predictor of SFTS in China [15, 19, 20], indicating that the disease is unlikely to occur at very elevated locations. Published studies [3–5, 11, 23] have also suggested that rural areas are likely to have higher levels SFTSV infection risk. The present study findings, showing a greater tendency of infections in rural areas, were well captured using farmland as an input variable. In Miyazaki, cases of SFTS in rural regions occurred in relatively lowland areas near rivers, which was well captured by jointly using altitude and farmland in the prediction model. Our modeling study findings lead us to conclude that the risk of SFTS is higher in geographic areas with a lower number of farms and at lower altitudes. The number of farms decrease as altitude increases, while altitude decreases as the location comes close to the ocean with greater number of farms than other locations, implying that areas with small number of farms and at mid-level altitudes may be at high risk of infection.

Considering geographic locations with a low density of farms, flatlands near hilly or mountainous locations along rivers were found to be hot spots of SFTSV infection. The implications of the present study results will greatly assist in future surveillance of both animals and vectors. For instance, local public health organizations regularly conduct surveillance of the tick distribution, and conduct sample collection and laboratory testing of *Ixodes* species to identify SFTSV infections [30]. The present study findings emphasize the need to selectively conduct frequent intensive ecological surveys in high-risk areas to identify the virus. Moreover, it is important to implement animal surveillance near high-risk areas, and to include surveys of wildlife species. The range of animal host species, including those acting as the interface between wildlife species and humans, could be better identified by conducting intensive surveys of animals in regions with high risk of SFTSV infections.

The prediction results of LR and GWLR were mostly comparable. This is owing to the shortage of mesh areas and also the small number of mesh areas with SFTS cases. Moreover, the size of Miyazaki Prefecture is small; in conducting 10 *×* 10-km mesh analysis with a substantial number of marginal areas, its small size made it difficult to demonstrate the better performance of GWLR compared with LR. However, GWLR outperformed LR in the sense of likelihood and also in the predictive performance evaluation, i.e., AUC. GWLR can be useful for reflecting local incidence data and identifying high-risk areas in a more meticulous manner.

The present study includes several limitations. First, the total number of mesh areas with SFTS was limited to 22 of 97 mesh units; thus, the resolution of prediction was limited (e.g., with a greater number of cases, one might even consider using a 1 *×* 1-km mesh unit). This may be regarded as imbalanced data, which is a threat to stable prediction in logistic regression, and the performance of model prediction is known to be significantly hindered by class imbalances. Although the observed imbalance ratio remained smaller than 4:1, the recognized problem would not be eased unless more data become available and the imbalances are resolved [45]. What must be remembered in this regard is that the observed patterns may not have adequately reflected the distribution of explanatory variables. Owing to a related reason (i.e., limited precision of mesh data), we were unable to incorporate an original (more concrete) variable, i.e., location close to rivers and in transitional zones between farms, into our prediction using a 10 *×* 10-km mesh unit. Second, the suspected locations of infection rested on interviews; considering the map scale of the original data, there could be measurement error with respect to the precision of the suggested geographic points at finer spatial resolutions. Third, whereas we focused on a binary outcome (i.e., presence or absence of SFTS cases), some mesh areas had already experienced multiple cases of SFTS. Owing to a small total number of cases, prediction of the number of cases is a future task to be performed. Fourth, we ignored climatological variables including temperature, humidity, and precipitation owing to the absence of dynamic (time-dependent) case data over geographic space [46]. Fifth, infection risk depends on both (i) human behaviors that lead to tick bites and (ii) the risk of infection among ticks (and animals) [47–49]. In the present study, we could not distinguish (i) and (ii) nor elucidate the detailed ecological mechanisms behind the observations [50]; this would require cross-disciplinary collaboration among epidemiologists and statisticians as well as entomologists, ecologists, veterinarians, and virologists.

Despite the number of important tasks that remain to be completed in future, in the present study, we successfully identified altitude and farmland as negative predictors of SFTS cases in Miyazaki, concluding that the risk of SFTS is high in geographic areas with decreased farmland and at middle altitudes. Our study findings not only indicate high-risk areas that are suitable for conducting ecological and animal surveys in the future, we also believe that it is critical to raise awareness among local residents in such areas about the existing risk of SFTS and to emphasize the importance of preventing tick bites at the local level.

## Conclusions

In the present study, we analyzed the spatial epidemiological risk of SFTS in Miyazaki, Japan, using 10 *×* 10-km mesh data and a GWLR model. By identifying altitude and farmland as negative predictors of SFTS cases in Miyazaki, we can conclude that the risk of SFTS is high in geographic areas where farmland begins to diminish and at locations where the altitude is not very high. Our findings suggest high-risk areas where future ecological and animal surveys are appropriate. We recommend raising awareness among local residents in high-risk areas about the risk of SFTS and emphasizing the importance of preventing tick bites.

## Additional file

Additional file 1: Datasets of mesh areas with observed cases of severe fever with thrombocytopenia syndrome (SFTS), with altitude and farmland, in Miyazaki, Japan (XLSX 13 kb)

## Data Availability

The occurrence of SFTS cases, altitude, and the proportion of farmland are available as Additional file 1.

## Abbreviations

AIC: Akaike Information Criterion
AUC: Area under the ROC curve
CFR: Case fatality risk
CI: Confidence interval
GWLR: Geographically weighted logistic regression
LR: Logistic regression
NPV: Negative predictive value
PPV: Positive predictive value
ROC: Receiver operating characteristic curve
SFTS: Severe fever with thrombocytopenia syndrome
SFTSV: Severe fever with thrombocytopenia syndrome virus
